# 

**DOI:** 10.1634/theoncologist.2012-MA-2

**Published:** 2012-11

## Abstract 1 – IDH and TET 2 Mutations in Human Leukemia

Ari Melnick, Cornell University, New York, New York

This presentation will describe the discovery of a new class of acute leukemias that share in common disruption of a novel gene regulatory mechanism that involves alterations in 5-hydroxymethylcytosine (5hmC). 5hmC is a newly discovered epigenetic mark that plays a key role in gene activation, whereas the better-known DNA methylation epigenetic mark is mostly associated with gene silencing. Somatic mutations in the IDH1, IDH2, and TET2 genes are the known causes of this leukemia subtype, but others may exist as well. Aberrant hydroxymethylation of genes in these patients is strongly linked to their aberrant expression. Although 5hmC is linked to DNA methylation, these two epigenetic marks tend to affect different regions of genes and may cooperate to induce the leukemia phenotype. Genetic lesions in IDH1 and IDH2 result in an aberrant enzymatic gain of function that disrupts 5hmC and DNA methylation through production of an oncometabolite called 2-hydroxyglutarate (2HG). 2HG disrupts 5hmC by inhibiting the function of TET2 and other enzymes. TET2 loss of function mutants partially reproduce the effects of IDH mutations, explaining why these two types of mutations are mutually exclusive in leukemia patients. Mice containing mutant IDH or TET2 in their bone marrow cells develop preleukemic syndromes with similar epigenetic defects as the human disease. Importantly, it is possible to design inhibitors that specifically inhibit mutant forms of IDH, which may restore normal programming of gene expression and kill leukemia cells. In addition, we will discuss novel biomarkers and therapeutic targets identified through epigenetic profiling of leukemias.

## Abstract 2 – Role of VEGFR and MET in Prostate Cancer and Bone Metastases

Matthew R. Smith, Massachusetts General Hospital Cancer Center, Harvard Medical School, Boston, Massachusetts

Bone metastases are the major cause of prostate cancer morbidity and mortality. VEGFR and MET signaling appears to play an important role in the development and progression of bone metastases. Over-expression of MET and/or HGF correlates with prostate cancer metastasis and disease recurrence. Osteoblasts and osteoclasts express MET and VEGFRs. Osteoclasts secrete HGF, suggesting that MET signaling may play a role in pathological bone remodeling in prostate cancer. MET and HGF expression are regulated by the androgen receptor. Androgen deprivation increases MET and HGF expression in tumor and/or stroma, suggesting that increased expression of MET and HGF may contribute to disease progression following androgen deprivation therapy. Agents that target primarily VEGF/VEGFR (bevacizumab, sunitinib) have reported limited activity in metastatic prostate cancer. In contrast, cabozantinib, a small molecule inhibitor of multiple targets including VEGFR and MET, has demonstrated remarkable early evidence of activity in metastatic castration-resistant prostate cancer. Cabozantinib is associated with high rates of partial or complete resolution of bone scans, improvements in measurable disease, decreased pain, and reductions in bone biomarkers and circulated tumor cells. Based on these observations, cabozantinib is now in phase 3 clinical development in prostate cancer.

## Abstract 3 – Targeting Quiescent Cancer Cells

Sridhar Ramaswamy, Massachusetts General Hospital Cancer Center, Harvard Medical School, Boston, Massachusetts

Human tumors contain many slowly proliferating cancer cells. We do not know how or why they arise, although these cells appear to be important mediators of drug resistance. We have recently found when cancer cells divide they use a highly conserved signaling pathway to induce the asymmetric degradation of AKT1 protein kinase in one daughter cell. This mechanism results in asymmetric cancer cell divisions that occur infrequently but continuously to produce slowly cycling AKT1^low^ cells that assume a unique proteomic, epigenomic, and transcriptional state. Interestingly, asymmetrically dividing cancer cells can produce symmetrically dividing progeny and vice versa, suggesting that asymmetric division is not the unique property of a specialized cancer cell subpopulation. Furthermore, we have found that the ability to divide asymmetrically in this way confers a selective advantage on cancer cells with broad implications for understanding how cancer cell populations proliferate, generate heterogeneity, evade treatment, and recur. We present this potentially new model of cancer cell division in molecular detail and discuss novel ideas for the molecular targeting of quiescent cancer cells in patients.

## Abstract 4 – Resistance to RAF Inhibition in BRAF Mutant Colorectal Cancer

Ryan B. Corcoran, Massachusetts General Hospital Cancer Center, Harvard Medical School, Boston, Massachusetts

BRAF V600 mutations occur in 10–15% of colorectal cancers (CRCs). Although RAF inhibitors have demonstrated response rates of ∼60–80% in BRAF mutant melanoma, they have shown only a ∼5% response rate in BRAF mutant CRC. Thus, there is a fundamental difference between BRAF mutant CRC and BRAF mutant melanoma, and understanding the basis for this difference would have important clinical implications. We observed that RAF inhibitors led to complete suppression of MAPK signaling in BRAF mutant melanomas, but only to transient inhibition of MAPK signaling in BRAF mutant CRC, likely explaining their insensitivity. We have identified several resistance mechanisms that lead to sustained MAPK signaling in the presence of RAF inhibitors in BRAF mutant CRC. BRAF amplification occurs in ∼10% of BRAF mutant colorectal cancers and can cause resistance to both RAF and MEK inhibitors. We have also found that EGFR mediates RAF inhibitor resistance in BRAF mutant CRC by reactivating the MAPK pathway through RAS and CRAF. BRAF mutant CRCs express higher levels of total and phosphorylated EGFR than BRAF mutant melanoma, perhaps explaining why they are prone to EGFR-mediated resistance. The therapeutic implications of these and other potential resistance mechanisms will be discussed. Elaboration of these resistance mechanisms has led to the development of novel targeted therapy combination strategies such as combined RAF/MEK inhibition, combined RAF/EGFR inhibition, and other strategies currently in development. The initial clinical experience with RAF/MEK inhibition as well as therapeutic strategies presently in development will also be discussed.

## Abstract 5 – Systematic Identification of Genomic Markers of Drug Sensitivity in Cancer Cells

Cyril Benes, Massachusetts General Hospital Cancer Center, Harvard Medical School, Boston, Massachusetts

Systematic molecular characterization of human tumors, in particular using next-generation sequencing, is yielding a comprehensive view of the heterogeneity of cancers. Moreover, it will soon be possible to characterize individual tumors at a similar level as part of a novel generation of diagnostics. To translate this knowledge into cancer care, there is a need to understand how variations in genome and other dimensions (epigenome, etc.) influence therapeutic responses and which genome variants might constitute good targets.

Building on the pioneering efforts of the National Cancer Institute using human tumor derived cell lines as tractable models for the study of drug sensitivity (the panel of 60 cell lines known as the NCI60), we have assembled a collection of 1,000 cell lines representative of the majority of adult and childhood cancers. We use this cell line collection to define molecular determinants of drug sensitivity in vitro as a first step towards defining biomarkers of drug response in patients. We have screened several hundred therapeutically relevant compounds and used multiple statistical approaches to model the relation between the cancer genome and drug sensitivity. Strikingly, our approach captures essentially all genotype–drug sensitivity associations reported to date and leveraged in the clinic through the most recent rationally designed therapies. We also identify a large number of novel correlates of drug sensitivity. Overall, our studies reveal many novel sensitizing and drug resistance correlates that could lead to the identification of biomarkers useful for the development and application of cancer therapeutics.

## Abstract 6 – Identifying Small Molecules that Overcome Differentiation Arrest in Acute Myeloid Leukemia

David Brian Sykes, Dana-Farber Cancer Institute, Massachusetts General Hospital Cancer Center, Boston, Massachusetts

Acute Myeloid Leukemia (AML) in adults is a devastating disease with a 5-year survival rate of 25%. We lack new treatments for AML, and the chemotherapy standard of care remains unchanged in thirty years. One success story in the treatment of AML has been the discovery of drugs that, rather than acting as cytotoxic agents, trigger the differentiation of leukemic blasts in the subset of patients with acute promyelocytic leukemia. However, differentiation therapy is not available for 90% of patients with AML.

Understanding and targeting the mechanism of differentiation arrest in AML has been under investigation for more than four decades. We developed a novel model system in which to identify small molecules that can promote myeloid differentiation. In this system, murine bone marrow cells were engineered to express the green fluorescent protein (GFP) specifically upon differentiation. These cells established the basis of a screen whereby the activity of small molecules to promote differentiation could be identified by simply assaying for green fluorescence using high-throughput flow cytometry.

In collaboration with the University of New Mexico Center for Molecular Discovery and The Broad Institute, we performed a screen of the ∼350,000 compounds in the NIH Molecular Libraries Small Molecule Repository. Here we present the details of our high-throughput flow cytometry system and preliminary identification of pro-differentiation agents in AML. If successful, we anticipate that one of these small molecules may offer insight into a mechanism for overcoming differentiation arrest, and may also translate into a novel, clinically relevant treatment for acute myeloid leukemia.

## Abstract 7 – Proteomic Approaches to Study Ubiquitination and Protein Stability

Andrew E.H. Elia, Massachusetts General Hospital Cancer Center, Harvard Medical School, Boston, Massachusetts

Cullin-RING ligases (CRLs) comprise the largest class of E3 ubiquitin ligases, and the identification of their substrates is important to understanding numerous processes, such as DNA damage and cell cycle signaling. Using pharmacologic Cullin inhibition, coupled with proteomic and genetic approaches, we have uncovered hundreds of proteins whose ubiquitination status or stabilities are regulated by CRLs. To proteomically assess changes in ubiquitination, we have utilized a peptide immunoaffinity-based approach and quantitative mass spectrometry. To genetically measure shifts in protein stability, we have employed a fluorescent reporter system that combines FACS with microarray deconvolution. Together, these methods have yielded many known CRL substrates as well as a multitude of novel putative substrates. These substrates are enriched for a property that indicates their location at highly connected nodes in protein interaction networks, representing critical connections between regulatory pathways and demonstrating the broad role of CRL ubiquitination in cellular biology.

## Abstract 8 – Functional Studies of the Isocitrate Dehydrogenase (IDH) Oncoprotein

Julie-Aurore Losman, Dana-Farber Cancer Institute, Harvard Medical School, Boston, Massachusetts

IDH1 and IDH2 mutants are common in several cancers, including myeloid leukemias, and overproduce the (*R*)-enantiomer of 2-hydroxyglutarate [(*R*)-2-HG]. (*R*)-2-HG is hypothesized to function by inhibiting the activity of diverse α-ketoglutarate-dependent enzymes that regulate the epigenetic landscape of cells, including the TET family of 5-methylcytosine hydroxylases and the jumonji-domain-containing family of histone demethylases. However, it has not been formally proven that (*R*)-2-HG is sufficient to transform cells, or that the putative transforming activity of (*R*)-2-HG is reversible. To investigate the role of (*R*)-2-HG in leukemogenesis, we developed a transformation assay using TF-1 cells, a growth factor-dependent human myeloid leukemia cell line. We found that a canonical IDH1 mutant, IDH1 R132H, is able to promote cytokine-independence and block differentiation of TF-1 cells. Interestingly, these effects could be recapitulated by a cell membrane-permeable form of (*R*)-2-HG, TFMB-(*R*)-2-HG, but not by TFMB-(*S*)-2-HG. This is noteworthy, as (*S*)-2-HG is a more potent inhibitor than (*R*)-2-HG of TET2, an enzyme that has been previously linked to the pathogenesis of IDH mutant leukemias. We found that this paradox relates to the ability of (*S*)-2-HG, but not (*R*)-2-HG, to inhibit the EglN prolyl hydroxylases, and found that inhibition of EglN1 is antithetical to transformation by mutant IDH. Furthermore, we found that transformation by TFMB-(*R*)-2-HG, and by IDH1 R132H, is reversible upon removal of (*R*)-2-HG. Our findings suggest that inhibitors that target (*R*)-2-HG production by mutant IDH, and inhibitors that target EglN1 activity, may have therapeutic efficacy in the treatment of myeloid leukemias that harbor IDH mutations.

## Abstract 9 – Smart T-Cell Vaccines

Carl H. June, University of Pennsylvania, Philadelphia, Pennsylvania

Chimeric antigen receptors (CARs) combine an antigen recognition domain of a specific antibody with a intracellular signaling domains into a single engineered protein. CD19 is an ideal target for CARs, since expression is restricted to normal and malignant B cells. In preclinical studies, inclusion of the CD137 (4–1BB) signaling domain results in remarkable antitumor activity and in vivo persistence of anti-CD19 CARs. We have previously reported dramatic in vivo expansion, persistence and anti-tumor activity of CAR modified autologous T cells targeted to CD19 (CART19 cells) in 3 patients with CLL with relatively short follow up (Porter, et al NEJM 2011; Kalos et al Sci Trans Med 2011). Currently, 10 patients have been treated with CART19 cells. There were 9 adults (median age, 65 years [range 51–78]) treated for relapsed, refractory and high risk CLL and one 7 year old treated for relapsed refractory pre-B cell ALL. CLL patients had received a median of 5 prior regimens, excluding single agent rituxumab (range, 2–10), and all had active disease at the time of cell infusion. The one patient with ALL was in 2nd chemorefractory relapse, having received Cytoxan/etoposide/clofarabine 6 weeks prior to infusion without response. A median of 7.5 × 108 total cells (range, 1.7–50) corresponding to 1.45 (range, 0.14–5.9 × 108) genetically modified cells were infused. In the patients in complete remission, CAR modified CART19 cells are detectable in the peripheral blood by flow cytometry at the most recent follow up for at least 2 years. All patients in CR have B cell aplasia. No patient with CR has progressed.

## Abstract 10 – Exosomes as Biomarkers of the Genetic Status of Brain Tumors

Xandra Breakefield, Massachusetts General Hospital Cancer Center, Harvard Medical School, Boston, Massachusetts

Nanosized membrane-bound vesicles (exosomes) are released in abundance from tumor cells and contain mutant mRNA sequences, elevated miRNAs, and amplified oncogene DNA characteristic of the tumors. Our team has evaluated the correlation between the nucleic acid content of exosomes and brain tumor cells in culture and in mouse tumor models. We have also explored the use of exosomes in serum/plasma and cerebral spinal fluid (CSF) from glioblastoma patients as biomarkers for the genetic status of individual tumors. We have detected EGFRvIII and IDH1 mutations in exosomes in these biofluids corresponding to mutations in glioma tumors, as well as elevated levels of exosomal miR21 - high in glioma tumors, and amplified c-Myc DNA sequences in exosomes from medulloblastoma tumors. We are exploring increasing the sensitivity of these biomarkers by enrichment of tumor-derived exosomes using microfluidic antibody capture, by high resolution sequence analysis and by diagnostic magnetic resonance to monitor tumor-associated proteins. Exosomes provide a potential means of monitoring dynamic changes in tumors in response to therapy.

## Abstract 11 – Targeting the PD-1 Pathway in Cancer Therapy

Suzanne L. Topalian, Johns Hopkins University School of Medicine, Sidney Kimmel Comprehensive Cancer Center, Baltimore, Maryland

Cancers harbor a multitude of mutations that can potentially be targeted by the immune system. However, cancer cells can co-opt normal immune tolerance mechanisms to evade immune destruction. The immunoglobulin-like molecule PD-L1 (B7-H1), expressed by many human cancers, creates a protective shield against immune attack. PD-L1 is a ligand for the programmed death-1 (PD-1) receptor normally expressed on activated T cells, and delivers an inhibitory signal to suppress antitumor immunity. Blocking the PD-1:PD-L1 interaction may rejuvenate antitumor immunity. Multicenter clinical trials of blocking antibodies against PD-1 or PD-L1 have been undertaken recently. In the first phase I trial of anti-PD-1 (BMS-936558/MDX-1106), patients with treatment-refractory metastatic melanoma, lung, kidney, colon, or prostate cancer received an intermittent dosing regimen. Durable objective responses were observed in patients with colon cancer, kidney cancer, and melanoma. Based on these results, a 296-patient phase I/II trial of biweekly outpatient administration of anti-PD-1, and the first-in-human trial of anti-PD-L1 (BMS-936559/MDX-1105), were initiated. Objective tumor regressions and prolonged stable disease were observed in patients with non-small cell lung cancer, renal cell cancer, or melanoma treated with either drug, highlighting the importance of the PD-1:PD-L1 pathway as a target for cancer therapy. Among patients with objective responses who were followed >1 year, responses lasted >1 year in 65% or 50% of patients (anti-PD-1 or anti-PD-L1, respectively), demonstrating durable treatment effects. Toxicities were generally manageable, and some were consistent with immune-based etiologies. Current investigations into pharmacodynamics and potential biomarkers of response are designed guide optimal clinical development of these drugs.

